# Supplementary material for: Methanolobus use unspecific methyltransferases to produce methane from dimethylsulphide in Baltic Sea sediments
Source: Microbiome. 2024 Jan 3;12:3. doi: 10.1186/s40168-023-01720-w (PMC10762971; doi:10.1186/s40168-023-01720-w)
Supplement: Supplementary file 2 — Additional file 1: Supplementary Figure 1. Map of Himmerfjärden and the Baltic Sea showing the three sampling stations H2, H3 and H5. The Stockholm University Baltic Sea Centre is located on the Askö Island. Inset map shows the entire Baltic Sea. Supplementary Figure 2. Average concentrations of DMS and methane in DMS-amended incubations from seven sediment layers (0-1 cm, 1-2 cm, 2-5 cm, 5-12 cm, 19-22 cm, 39-43 cm, 60-65 cm). Black lines: DMS; Red lines: Methane. Supplementary Figure 3. Average sulfate concentrations at the start and the end of the incubation period in the samples D1: 0-1 cm; D2: 1-2 cm; D3: 2-5 cm; D4: 9-12 cm; D5: 19-22 cm; D6: 39-43 cm; D7: 60-65 cm. Supplementary Figure 4. Mean copy number of the mcrA gene per gram of wet sediment in the original and DMS-amended sediments. Error bars represent standard error above and below the average of three replicates. Supplementary Figure 5. Presence and absence of the genes involved in methane production in the four Methanolobus MAGs constructed using the metagenomics datasets. (a) Distinct genes involved in acetoclastic, hydrogenotrophic and methylotrophic methanogenesis pathways; (b) Genes common to all methanogenesis pathways. Supplementary Figure 6. Heatmap showing the normalised copy numbers of the genes common in all methanogenesis pathways. (a) Metagenomics datasets; (b) Metatranscriptomics datasets. CPM: Copies per million reads; FPKM: fragments per kilobase of gene per million reads. Supplementary Figure 7. Heatmap showing the normalised copy numbers of the genes involved in acetoclastic and hydrogenotrophic methanogenesis pathways. (a) Metatranscriptomics datasets; (B) Metagenomics datasets. FPKM: fragments per kilobase of gene per million reads. CPM: Copies per million reads. Supplementary Table 1. Spearman’s rank correlation coefficients (rs) between total DMS consumed, total methane and CO2 produced, depth, initial and end point sulfate amounts and the first two principal coordinates obtaine [file 40168_2023_1720_MOESM1_ESM.pdf]

Methanolobus use unspecific methyltransferases to produce methane from dimethylsulfide in Baltic Sea sediments

S. L. Tsola, Y. Zhu, Y. Chen, I. A. Sanders, C. K. Economou,

V. Brüchert and Ö. Eyice

Supplementary Material

Metagenomics Analysis

The metagenomics data were processed to remove contamination, adapter sequences and low-quality reads. After filtering, the reads were assembled with metaSPAdes (3.13.0; Nurk et al., 2017) and mapped back to contigs using BBMap (38.44; Bushnell, 2014). The assembled contigs were then used for feature prediction. The workflow predicted noncoding RNA genes (tRNAs: tRNAscan-SE 2.0.6; Chan and Lowe, 2019, other noncoding RNA: cmsearch from the INFERNAL 1.1.3 package using the Rfam 13.0 database; Nawrocki and Eddy, 2013; Kalvari et al., 2018), clustered regularly interspaced short palindromic repeats (CRISPR; JGI modified version of CRT-CLI 1.2; Bland et al., 2007) and protein-coding genes (CDSs; Prodigal 2.6.3 and GeneMarkS-2 1.07; Hyatt et al., 2010; Lomsadze et al., 2018).

Functional annotation consisted of associating CDSs with KEGG orthology (KO) terms and Enzyme Commission (EC) numbers (lastal 1066 - LAST package; Kiełbasa et al., 2011), clusters of orthologous genes (COGs; Galperin et al., 2015) assignments, SMART (01_06_2016; Letunic and Bork, 2018) domains, SUPERFAMILY (1.75; Gough et al., 2001)

assignments, CATH-FunFam (4.2.0; Sillitoe et al., 2019) annotations and Pfam (30; Finn et al., 2016), and TIGRFAM (15.0; Haft et al., 2013) annotations using HMMER 3.1b2 (Mistry et al., 2013). Taxonomic annotation of the CDSs was completed using the best LAST hits during the KO term assignment during functional annotation.

MetaBAT (2.12.1; Kang et al., 2015) generated genome bins using the assembled contigs. The genome bins were then analysed for contamination removal. Genome completion and contamination estimates were determined using CheckM (1.0.12; Parks et al., 2015). Using the contaminations estimates alongside rRNA and tRNA information from the annotation steps, the genome bins were assigned high quality (HQ) or medium quality (MQ) values per the Minimum Information about a Metagenome-Assembled Genome (MIMAG) standards (Bowers et al., 2017). For each bin, two methods were used to determine their phylogenetic lineage, an internal Integrated Microbial Genome (IMG; Chen et al., 2021) program and GTDB-tk (0.2.2; Chaumeil et al., 2020).

References

Bland, C., Ramsey, T. L., Sabree, F., Lowe, M., Brown, K., Kyrpides, N. C., & Hugenholtz, P. (2007). CRISPR Recognition Tool (CRT): A tool for automatic detection of clustered regularly interspaced palindromic repeats. *BMC Bioinformatics*, *8*, 1–8.

Bowers, R. M., Kyrpides, N. C., Stepanauskas, R., Harmon-Smith, M., Doud, D., Reddy, T. B. K., Schulz, F., Jarett, J., Rivers, A. R., Eloe-Fadrosh, E. A., Tringe, S. G., Ivanova, N. N., Copeland, A., Clum, A., Becraft, E. D., Malmstrom, R. R., Birren, B., Podar, M., Bork, P., … Woyke, T. (2017). Minimum information about a single amplified genome (MISAG) and a metagenome-assembled genome (MIMAG) of bacteria and archaea. *Nature Biotechnology*, *35*(8), 725–731.

Bushnell, B. (2014). *BBMap: A Fast, Accurate, Splice-Aware Aligner*. https://www.osti.gov/biblio/1241166

Chan, P., & Lowe, T. (2019). tRNAscan-SE: searching for tRNA genes. *Gene Prediction*, *1962*, 1–21.

Chaumeil, P. A., Mussig, A. J., Hugenholtz, P., & Parks, D. H. (2020). GTDB-Tk: A toolkit to classify genomes with the genome taxonomy database. *Bioinformatics*, *36*(6), 1925–1927.

Chen, I. M. A., Chu, K., Palaniappan, K., Ratner, A., Huang, J., Huntemann, M., Hajek, P., Ritter, S., Varghese, N., Seshadri, R., Roux, S., Woyke, T., Eloe-Fadrosh, E. A., Ivanova, N. N., & Kyrpides, N. C. (2021). The IMG/M data management and analysis system v.6.0: New tools and advanced capabilities. *Nucleic Acids Research*, *49*(D1), D751–D763.

Finn, R. D., Coggill, P., Eberhardt, R. Y., Eddy, S. R., Mistry, J., Mitchell, A. L., Potter, S. C., Punta, M., Qureshi, M., Sangrador-Vegas, A., Salazar, G. A., Tate, J., & Bateman, A. (2016). The Pfam protein families database: Towards a more sustainable future. *Nucleic Acids Research*, *44*(D1), D279–D285.

Galperin, M. Y., Makarova, K. S., Wolf, Y. I., & Koonin, E. v. (2015). Expanded Microbial genome coverage and improved protein family annotation in the COG database. *Nucleic Acids Research*, *43*(D1), D261–D269.

Gough, J., Karplus, K., Hughey, R., & Chothia, C. (2001). Assignment of homology to genome sequences using a library of hidden Markov models that represent all proteins of known structure. *Journal of Molecular Biology*, *313*(4), 903–919.

Haft, D. H., Selengut, J. D., Richter, R. A., Harkins, D., Basu, M. K., & Beck, E. (2013). TIGRFAMs and genome properties in 2013. *Nucleic Acids Research*, *41*(D1), 387–395.

Hyatt, D., Chen, G.-L., LoCascio, P. F., Land, M. L., Larimer, F. W., & Hauser, LorenJ. (2010). Prodigal: prokaryotic gene recognition and translation initiation site identification. *BMC Bioinformatics*, *11*(119), 1–11.

Kalvari, I., Argasinska, J., Quinones-Olvera, N., Nawrocki, E. P., Rivas, E., Eddy, S. R., Bateman, A., Finn, R. D., & Petrov, A. I. (2018). Rfam 13.0: Shifting to a genome-centric resource for non-coding RNA families. *Nucleic Acids Research*, *46*(D1), D335–D342.

Kang, D. D., Froula, J., Egan, R., & Wang, Z. (2015). MetaBAT, an efficient tool for accurately reconstructing single genomes from complex microbial communities. *PeerJ*, *2015*(8), 1–15.

Kiełbasa, S. M., Wan, R., Sato, K., Horton, P., & Frith, M. C. (2011). Adaptive seeds tame genomic sequence comparison. *Genome Research*, *21*(3), 487–493.

Letunic, I., & Bork, P. (2018). 20 years of the SMART protein domain annotation resource. *Nucleic Acids Research*, *46*(D1), D493–D496.

Lomsadze, A., Gemayel, K., Tang, S., & Borodovsky, M. (2018). Modelling leaderless transcription and atypical genes results in more accurate gene prediction in prokaryotes. *Genome Research*, *28*(7), 1079–1089.
Mistry, J., Finn, R. D., Eddy, S. R., Bateman, A., & Punta, M. (2013). Challenges in homology search: HMMER3 and convergent evolution of coiled-coil regions. *Nucleic Acids Research*, *41*(12).

Nawrocki, E. P., & Eddy, S. R. (2013). Infernal 1.1: 100-fold faster RNA homology searches. *Bioinformatics*, *29*(22), 2933–2935.

Nurk, S., Meleshko, D., Korobeynikov, A., & Pevzner, P. A. (2017). MetaSPAdes: A new versatile metagenomic assembler. *Genome Research*, *27*(5), 824–834.

Parks, D. H., Imelfort, M., Skennerton, C. T., Hugenholtz, P., & Tyson, G. W. (2015). CheckM: Assessing the quality of microbial genomes recovered from isolates, single cells, and metagenomes. *Genome Research*, *25*(7), 1043–1055.

Sillitoe, I., Dawson, N., Lewis, T. E., Das, S., Lees, J. G., Ashford, P., Tolulope, A., Scholes, H. M., Senatorov, I., Bujan, A., Ceballos Rodriguez-Conde, F., Dowling, B., Thornton, J., & Orengo, C. A. (2019). CATH: Expanding the horizons of structure-based functional annotations for genome sequences. *Nucleic Acids Research*, *47*(D1), D280–D284.

Metatranscriptomics Analysis

The metatranscriptomics data were processed to remove contamination, adapter sequences and low-quality reads using BBDuk (38.87; Bushnell, 2014).

After filtering, the reads were assembled with MEGAHIT (1.2.9; Li et al., 2015), and the final assembly and coverage information were generated using BBMap (38.86; Bushnell, 2014). The assembled contigs were then used for structural annotation. The workflow predicted noncoding RNA genes (tRNAs: tRNAscan-SE 2.0.7; Chan and Lowe, 2019, other noncoding RNA: cmsearch from the INFERNAL 1.1.3 package using the Rfam 13.0 database; Nawrocki and Eddy, 2013; Kalvari et al., 2018), clustered regularly interspaced short palindromic repeats (CRISPR; JGI modified version of CRT-CLI 1.2; Bland et al., 2007) and protein-coding genes (CDSs; Prodigal 2.6.3 and GeneMark.hmm-2 (1.05; Hyatt et al., 2010; Lomsadze et al., 2018).

Functional annotation consisted of associating CDSs with KO terms and Enzyme Commission (EC) numbers (lastal 1066 - LAST package; Kiełbasa et al., 2011), COG (2003; Galperin et al., 2015) assignments, SMART (01_06_2016; Letunic and Bork, 2018) domains,

SUPERFAMILY (1.75; (Gough et al., 2001) assignments, CATH-FunFam (4.2.0; Sillitoe et al., 2019) annotations, Pfam (30; Finn et al., 2016), and TIGRFAM (15.0; Haft et al., 2013) annotations using HMMER 3.1b2 (Mistry et al., 2013). Taxonomic annotation of the CDSs was completed using the best LAST hits during the KO term assignment during functional annotation.

References

Bland, C., Ramsey, T. L., Sabree, F., Lowe, M., Brown, K., Kyrpides, N. C., & Hugenholtz, P. (2007). CRISPR Recognition Tool (CRT): A tool for automatic detection of clustered regularly interspaced palindromic repeats. *BMC Bioinformatics*, *8*, 1–8.

Bushnell, B. (2014). *BBMap: A Fast, Accurate, Splice-Aware Aligner*. https://www.osti.gov/biblio/1241166

Chan, P., & Lowe, T. (2019). tRNAscan-SE: searching for tRNA genes. *Gene Prediction*, *1962*, 1–21.

Finn, R. D., Coggill, P., Eberhardt, R. Y., Eddy, S. R., Mistry, J., Mitchell, A. L., Potter, S. C., Punta, M., Qureshi, M., Sangrador-Vegas, A., Salazar, G. A., Tate, J., & Bateman, A. (2016). The Pfam protein families database: Towards a more sustainable future. *Nucleic Acids Research*, *44*(D1), D279–D285.

Galperin, M. Y., Makarova, K. S., Wolf, Y. I., & Koonin, E. v. (2015). Expanded Microbial genome coverage and improved protein family annotation in the COG database. *Nucleic Acids Research*, *43*(D1), D261–D269.

Gough, J., Karplus, K., Hughey, R., & Chothia, C. (2001). Assignment of homology to genome sequences using a library of hidden Markov models that represent all proteins of known structure. *Journal of Molecular Biology*, *313*(4), 903–919.

Haft, D. H., Selengut, J. D., Richter, R. A., Harkins, D., Basu, M. K., & Beck, E. (2013). TIGRFAMs and genome properties in 2013. *Nucleic Acids Research*, *41*(D1), 387–395.

Hyatt, D., Chen, G.-L., LoCascio, P. F., Land, M. L., Larimer, F. W., & Hauser, LorenJ. (2010). Prodigal: prokaryotic gene recognition and translation initiation site identification. *BMC Bioinformatics*, *11*(119), 1–11.

Kalvari, I., Argasinska, J., Quinones-Olvera, N., Nawrocki, E. P., Rivas, E., Eddy, S. R., Bateman, A., Finn, R. D., & Petrov, A. I. (2018). Rfam 13.0: Shifting to a genome-centric resource for non-coding RNA families. *Nucleic Acids Research*, *46*(D1), D335–D342.
Kiełbasa, S. M., Wan, R., Sato, K., Horton, P., & Frith, M. C. (2011). Adaptive seeds tame genomic sequence comparison. *Genome Research*, *21*(3), 487–493.

Letunic, I., & Bork, P. (2018). 20 years of the SMART protein domain annotation resource. *Nucleic Acids Research*, *46*(D1), D493–D496.

Li, D., Liu, C. M., Luo, R., Sadakane, K., & Lam, T. W. (2015). MEGAHIT: An ultra-fast single-node solution for large and complex metagenomics assembly via succinct de Bruijn graph. *Bioinformatics*, *31*(10), 1674–1676.

Lomsadze, A., Gemayel, K., Tang, S., & Borodovsky, M. (2018). Modelling leaderless transcription and atypical genes results in more accurate gene prediction in prokaryotes. *Genome Research*, *28*(7), 1079–1089.

Mistry, J., Finn, R. D., Eddy, S. R., Bateman, A., & Punta, M. (2013). Challenges in homology search: HMMER3 and convergent evolution of coiled-coil regions. *Nucleic Acids Research*, *41*(12).

Nawrocki, E. P., & Eddy, S. R. (2013). Infernal 1.1: 100-fold faster RNA homology searches. *Bioinformatics*, *29*(22), 2933–2935.

Sillitoe, I., Dawson, N., Lewis, T. E., Das, S., Lees, J. G., Ashford, P., Tolulope, A., Scholes, H. M., Senatorov, I., Bujan, A., Ceballos Rodriguez-Conde, F., Dowling, B., Thornton, J., & Orengo, C. A. (2019). CATH: Expanding the horizons of structure-based functional annotations for genome sequences. *Nucleic Acids Research*, *47*(D1), D280–D284.

Codes for the analysis on Github:

https://github.com/StephTs/Methanolobus_and_DMS

https://doi.org/10.5281/zenodo.8426632

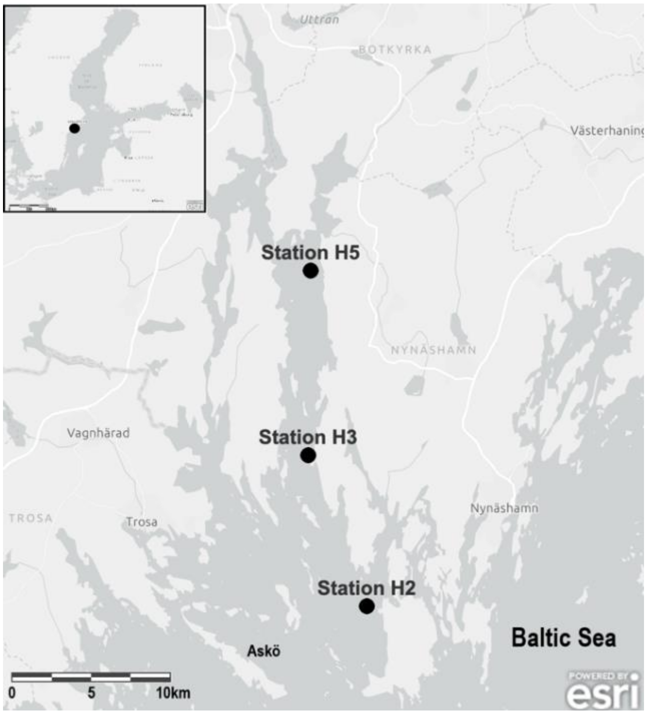

Supplementary Figure 1. Map of Himmerfjärden and the Baltic Sea showing the three sampling stations H2, H3 and H5. The Stockholm University Baltic Sea Centre is located on the Askö Island. Inset map shows the entire Baltic Sea.

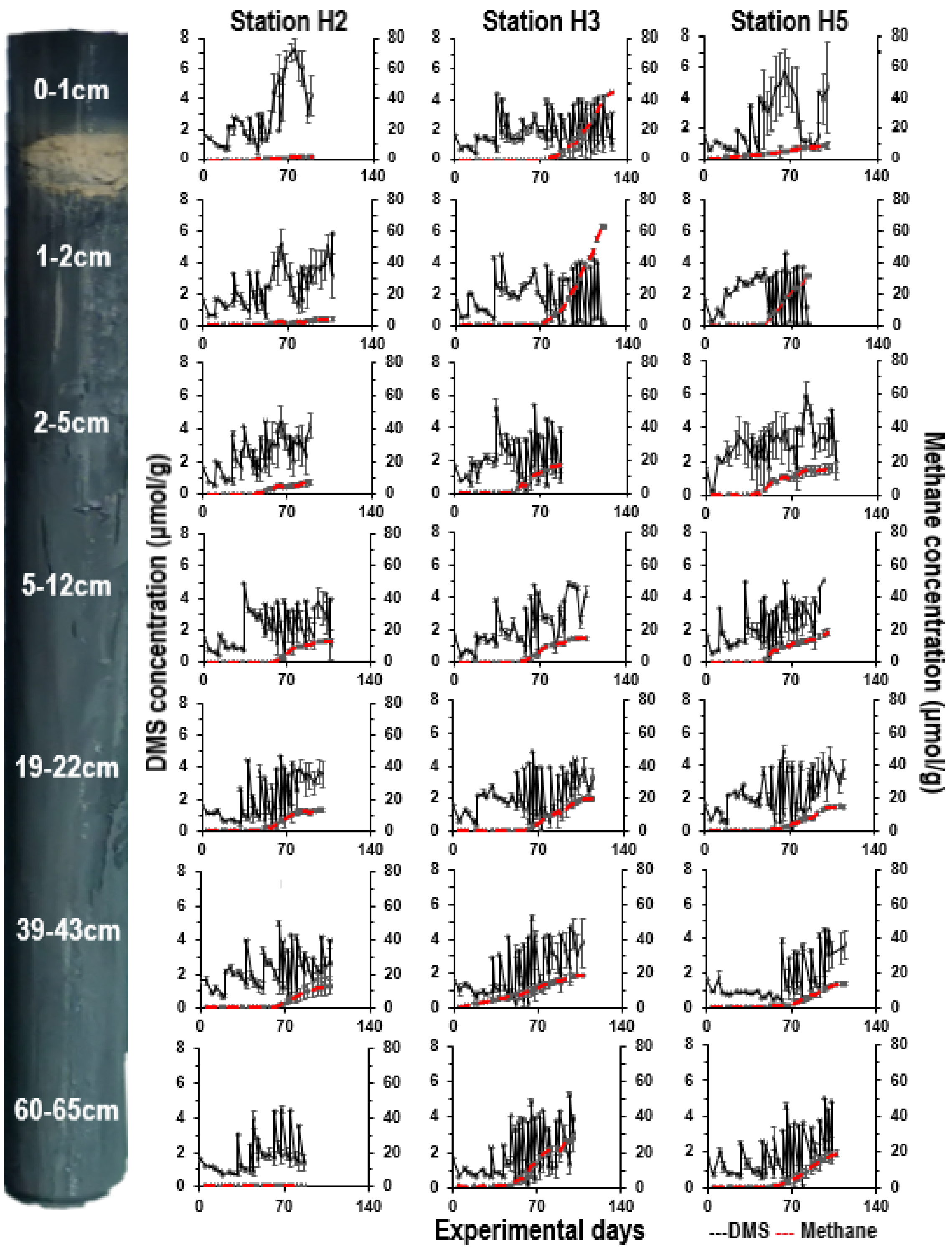

Supplementary Figure 2. Average concentrations of DMS and methane in DMS-amended incubations from seven sediment layers (0-1 cm, 1-2 cm, 2-5 cm, 5-12 cm, 19-22 cm, 39-43 cm, 60-65 cm). Black lines: DMS; Red lines: Methane

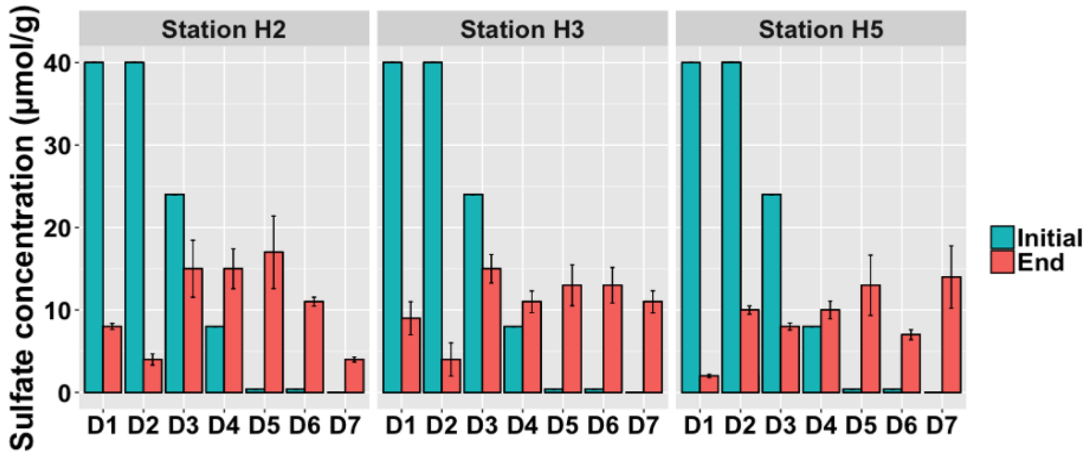

Supplementary Figure 3. Average sulfate concentrations at the start and the end of the incubation period in the samples D1: 0-1 cm; D2: 1-2 cm; D3: 2-5 cm; D4: 9-12 cm; D5: 19-22 cm; D6: 39-43 cm; D7: 60-65 cm.

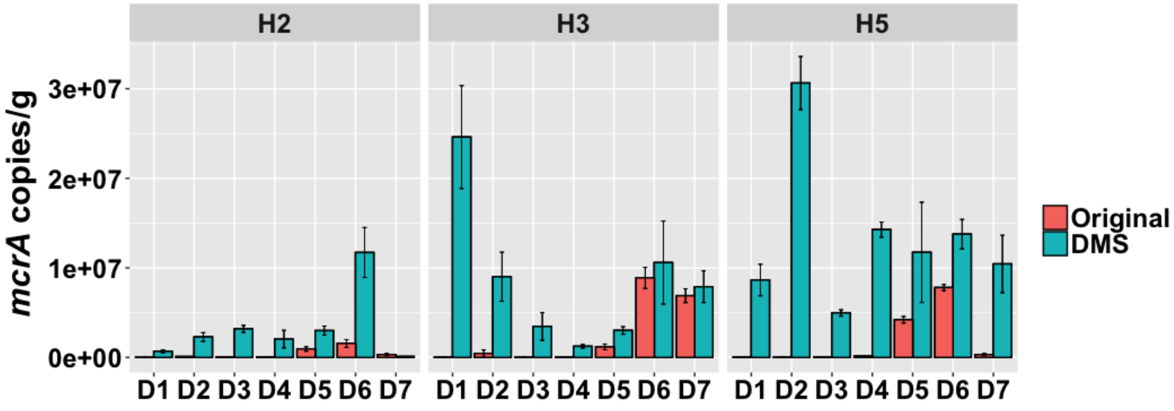

Supplementary Figure 4. Mean copy number of the *mcrA* gene per gram of wet sediment in the original and DMS-amended sediments. Error bars represent standard error above and below the average of three replicates.

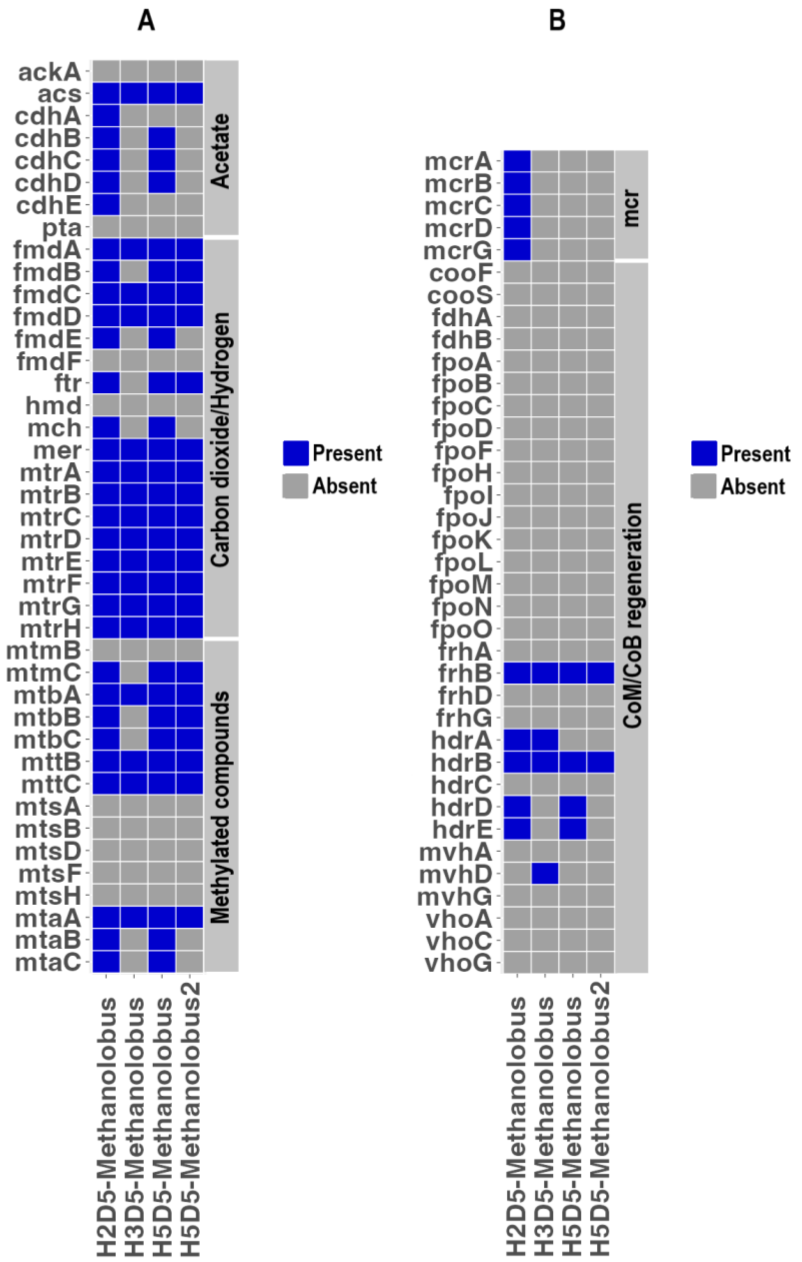

Supplementary Figure 5. Presence and absence of the genes involved in methane production in the four *Methanolobus* MAGs constructed using the metagenomics datasets. (a) Distinct genes involved in acetoclastic, hydrogenotrophic and methylotrophic methanogenesis pathways; (b) Genes common to all methanogenesis pathways.

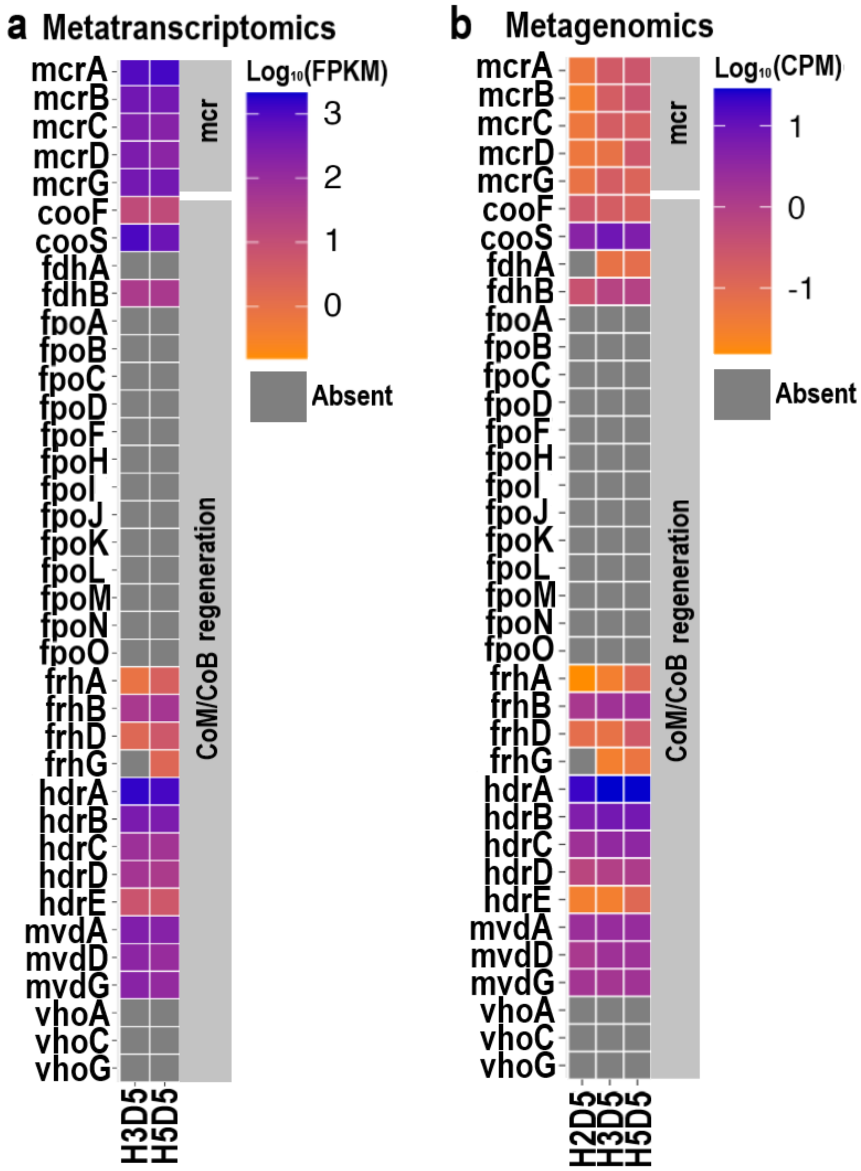

Supplementary Figure 6. Heatmap showing the normalised copy numbers of the genes common in all methanogenesis pathways. (a) Metagenomics datasets; (b) Metatranscriptomics datasets. CPM: Copies per million reads; FPKM: fragments per kilobase of gene per million reads.

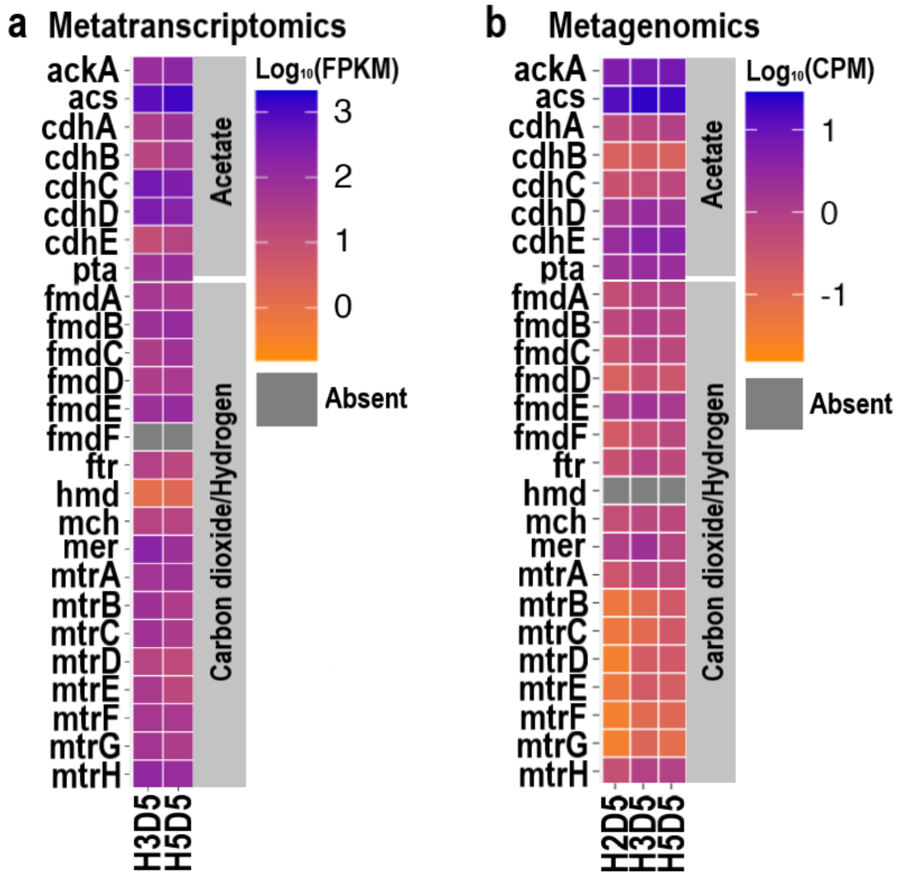

Supplementary Figure 7. Heatmap showing the normalised copy numbers of the genes involved in acetoclastic and hydrogenotrophic methanogenesis pathways. (a) Metatranscriptomics datasets; (B) Metagenomics datasets. FPKM: fragments per kilobase of gene per million reads. CPM: Copies per million reads.

Supplementary Table 1. Spearman's rank correlation coefficients (r_S) between total DMS consumed, total methane and CO_2 produced, depth, initial and end point sulfate amounts and the first two principal coordinates obtained by the *mcrA* sequence analysis. Statistically significant values are in bold. ***: p<0.001; **: p<0.01; *: p<0.05.

Spearman's rank correlation (r_S)	PCo1	PCo2
DMS consumed (µmol)	**0.85*** **	-0.17
Methane produced (µmol)	**0.84*** **	0.13
CO_2 produced (µmol)	**0.54*** **	-0.03
Initial sulfate (µmol)	**0.23***	**-0.56*** **
End sulfate (µmol)	**0.19***	**-0.42*** **
Depth (cm)	**-0.20***	**0.55*** **

Supplementary Table 2. The list of 78 methanogenesis-related genes searched within metagenomes and metatranscriptomes.

Genes	Metabolic Pathway	Full_Name	GeneCode
ackA	Methanogenesis (Acetate)	Acetate_kinase	K00925
acs	Methanogenesis (Acetate)	Acetyl-CoA synthetase	EG11448
cdhA	Methanogenesis (Acetate)	anaerobic carbon-monoxide dehydrogenase	K00192
cdhB	Methanogenesis (Acetate)	anaerobic carbon-monoxide dehydrogenase	K00195
cdhC	Methanogenesis (Acetate)	acetyl-CoA decarbonylase/synthase	K00193
cdhD	Methanogenesis (Acetate)	acetyl-CoA decarbonylase/synthase	K00194
cdhE	Methanogenesis (Acetate)	acetyl-CoA decarbonylase/synthase	K00197
pta	Methanogenesis (Acetate)	phosphate acetyltransferase	K00625
fmdA	Methanogenesis (CO2)	formylmethanofuran dehydrogenase	K00200
fmdB	Methanogenesis (CO2)	formylmethanofuran dehydrogenase	K00201
fmdC	Methanogenesis (CO2)	formylmethanofuran dehydrogenase	K00202
fmdD	Methanogenesis (CO2)	formylmethanofuran dehydrogenase	K00203
fmdE	Methanogenesis (CO2)	formylmethanofuran dehydrogenase	K11261
fmdF	Methanogenesis (CO2)	4Fe-4S ferredoxin	K00205
ftr	Methanogenesis (CO2)	formylmethanofuran--tetrahydromethanopterin N-formyltransferase	K00672
hmd	Methanogenesis (CO2)	5,10-methenyltetrahydromethanopterin hydrogenase	K13942
mch	Methanogenesis (CO2)	methenyltetrahydromethanopterin cyclohydrolase	K01499
mer	Methanogenesis (CO2)	5,10-methylenetetrahydromethanopterin reductase	K00320
mtrA	Methanogenesis (CO2)	tetrahydromethanopterin S-methyltransferase subunit A	K00577
mtrB	Methanogenesis (CO2)	tetrahydromethanopterin S-methyltransferase subunit B	K00578
mtrC	Methanogenesis (CO2)	tetrahydromethanopterin S-methyltransferase subunit C	K00579
mtrD	Methanogenesis (CO2)	tetrahydromethanopterin S-methyltransferase subunit D	K00580
mtrE	Methanogenesis (CO2)	tetrahydromethanopterin S-methyltransferase subunit E	K00581
mtrF	Methanogenesis (CO2)	tetrahydromethanopterin S-methyltransferase subunit F	K00582
mtrG	Methanogenesis (CO2)	tetrahydromethanopterin S-methyltransferase subunit G	K00583
mtrH	Methanogenesis (CO2)	tetrahydromethanopterin S-methyltransferase subunit H	K00584
mtmB	Methanogenesis (Methylamine)	methylamine---corrinoid protein Co-methyltransferase	K16176
mtmC	Methanogenesis (Methylamine)	monomethylamine corrinoid protein	K16177
mtbA	Methanogenesis (Dimethylamine)	[methyl-Co(III) methylamine-specific corrinoid protein]:coenzyme M methyltransferase	K14082
mtbB	Methanogenesis (Dimethylamine)	dimethylamine---corrinoid protein Co-methyltransferase	K16178
mtbC	Methanogenesis (Trimethylamine)	dimethylamine corrinoid protein	K16179
mttB	Methanogenesis (Trimethylamine)	trimethylamine---corrinoid protein Co-methyltransferase	K14083
mttC	Methanogenesis (Trimethylamine)	trimethylamine corrinoid protein	K14084
mtsA	Methanogenesis (Dimethylsulfide, methanethiol, methylpropionate)	methylthiol:coenzyme M methyltransferase	K16954
mtsB	Methanogenesis (Dimethylsulfide, methanethiol, methylpropionate)	methylated-thiol--corrinoid protein	K16955
mtsD	Methanogenesis (Dimethylsulfide, methanethiol, methylpropionate)	methyltransferase cognate corrinoid protein [Methanosarcina acetivorans C2A]	MA0859
mtsF	Methanogenesis (Dimethylsulfide, methanethiol, methylpropionate)	cobalamin-dependent protein [Methanosarcina acetivorans C2A]	MA4384
mtsH	Methanogenesis (Dimethylsulfide, methanethiol, methylpropionate)	cobalamin-dependent protein [Methanosarcina acetivorans C2A]	MA4558
mtaA	Methanogenesis (Methanol)	[methyl-Co(III) methanol/glycine betaine-specific corrinoid protein]:coenzyme M methyltransferase	K14080
mtaB	Methanogenesis (Methanol)	methanol---5-hydroxybenzimidazolylcobamide Co-methyltransferase	K04480
mtaC	Methanogenesis (Methanol)	methanol corrinoid protein	K14081
mcrA	Coenzyme M reduction to methane	methyl-coenzyme M reductase alpha subunit	K00399
mcrB	Coenzyme M reduction to methane	methyl-coenzyme M reductase beta subunit	K00401
mcrC	Coenzyme M reduction to methane	methyl-coenzyme M reductase subunit C	K03421
mcrD	Coenzyme M reduction to methane	methyl-coenzyme M reductase subunit D	K03422
mcrG	Coenzyme M reduction to methane	methyl-coenzyme M reductase subunit gamma	K00402
cooF	Coenzyme B/Coenzyme M regeneration	anaerobic carbon-monoxide dehydrogenase iron sulfur subunit	K00196
cooS	Coenzyme B/Coenzyme M regeneration	anaerobic carbon-monoxide dehydrogenase catalytic subunit	K00198
fdhA	Coenzyme B/Coenzyme M regeneration	glutathione-independent formaldehyde dehydrogenase	K00148
fdhB	Coenzyme B/Coenzyme M regeneration	formate dehydrogenase (coenzyme F420) beta subunit	K00125
fpoA	Coenzyme B/Coenzyme M regeneration	F420H2 dehydrogenase subunit A	K22158
fpoB	Coenzyme B/Coenzyme M regeneration	F420H2 dehydrogenase subunit B	K22159
fpoC	Coenzyme B/Coenzyme M regeneration	F420H2 dehydrogenase subunit C	K22160
fpoD	Coenzyme B/Coenzyme M regeneration	F420H2 dehydrogenase subunit D	K22161
fpoF	Coenzyme B/Coenzyme M regeneration	F420H2 dehydrogenase subunit F	K22162
fpoH	Coenzyme B/Coenzyme M regeneration	F420H2 dehydrogenase subunit H	K22163
fpoI	Coenzyme B/Coenzyme M regeneration	F420H2 dehydrogenase subunit I	K22164
fpoJ	Coenzyme B/Coenzyme M regeneration	F420H2 dehydrogenase subunit J	K22165
fpoK	Coenzyme B/Coenzyme M regeneration	F420H2 dehydrogenase subunit K	K22166
fpoL	Coenzyme B/Coenzyme M regeneration	F420H2 dehydrogenase subunit L	K22167
fpoM	Coenzyme B/Coenzyme M regeneration	F420H2 dehydrogenase subunit M	K22168
fpoN	Coenzyme B/Coenzyme M regeneration	F420H2 dehydrogenase subunit N	K22169
fpoO	Coenzyme B/Coenzyme M regeneration	F420H2 dehydrogenase subunit O	K22170
frhA	Coenzyme B/Coenzyme M regeneration	coenzyme F420 hydrogenase subunit alpha	K00440
frhB	Coenzyme B/Coenzyme M regeneration	coenzyme F420 hydrogenase subunit beta	K00441
frhD	Coenzyme B/Coenzyme M regeneration	coenzyme F420 hydrogenase subunit delta	K00442
frhG	Coenzyme B/Coenzyme M regeneration	coenzyme F420 hydrogenase subunit gamma	K00443
hdrA	Coenzyme B/Coenzyme M regeneration	heterodisulfide reductase	K03388
hdrB	Coenzyme B/Coenzyme M regeneration	heterodisulfide reductase	K03389
hdrC	Coenzyme B/Coenzyme M regeneration	heterodisulfide reductase	K03390
hdrD	Coenzyme B/Coenzyme M regeneration	heterodisulfide reductase	K08264
hdrE	Coenzyme B/Coenzyme M regeneration	heterodisulfide reductase	K08265
mvdA	Coenzyme B/Coenzyme M regeneration	F420-non-reducing hydrogenase large subunit	K14126
mvdD	Coenzyme B/Coenzyme M regeneration	F420-non-reducing hydrogenase iron-sulfur subunit	K14127
mvdG	Coenzyme B/Coenzyme M regeneration	F420-non-reducing hydrogenase small subunit	K14128
vhoA	Coenzyme B/Coenzyme M regeneration	methanophenazine hydrogenase, large subunit	K14068
vhoC	Coenzyme B/Coenzyme M regeneration	methanophenazine hydrogenase, cytochrome b subunit	K14069
vhoG	Coenzyme B/Coenzyme M regeneration	methanophenazine hydrogenase	K14070

Supplementary Table 3. Metagenome assembled genomes (MAGs) constructed from metagenome datasets from each sampling station at 19-22 cm of depth. Quality is based on the MIMAG (Bowers et al., 2017). Comp: Completeness; Cont: Contamination. Methanogen MAGs are in bold.

Site	Kingdom	Organism	Comp	Cont	Bases	Genes	Quality
**Station H2**	Bacteria	*Sideroxydans (Nitromonadales)*	99.37%	0.03%	2,570,235	2,553	Medium
	Bacteria	*Sulfuricella (Nitrosomonadales)*	98.66%	1.18%	3,054,443	3,112	Medium
	Bacteria	*Sulfurimonas (Campylobacterales)*	98.36%	2.12%	2,640,566	2,642	Medium
	Bacteria	*Sulfurivermis (Thiohalomonadales)*	97.89%	0.94%	3,721,594	3,717	Medium
	Bacteria	*Thiobacillus (Burkholderiales)*	97.37%	4.37%	3,104,052	3,228	Medium
	Bacteria	*SLDE01 (Thiohalomonadales)*	91.02%	2.11%	2,870,290	2,863	Medium
	Bacteria	*SPDF01 (Gemmatimonadales)*	84.84%	7.24%	2,284,254	2,441	Medium
	Bacteria	*Mor1 (Acidobacteriota)*	84.22%	4.70%	3,172,399	3,142	Medium
	Bacteria	*UBA2270 (Desulfobulbales)*	83.70%	0%	2,242,279	2,255	Medium
	Bacteria	*M0040 (Desulfuromonadales)*	78.40%	1.45%	2,612,526	2,764	Medium
	Bacteria	*UBA9959 (Elusimicrobiales)*	66.07%	1.71%	2,008,712	2,008	Medium
	Bacteria	*UBA2258*	64.75%	0.20%	2,201,006	2,164	Medium
	Bacteria	*GWC2-71-9 (Gemmatimonadales)*	62.74%	4.50%	1,941,363	2,018	Medium
	Bacteria	*Lutibacter (Flavobacteriales)*	61.48%	2.74%	1,850,908	1,961	Medium
	Bacteria	*Pontiella (Kiritimatiellales)*	58.46%	0.54%	2,736,742	2,701	Medium
	Bacteria	*CG2-30-66-27 (MBNT15)*	52.66%	0.84%	982,015	1,132	Medium
	Bacteria	*Ignavibacteriaceae (Ignavibacteriales)*	52.42%	2.33%	1,514,737	1,573	Medium
	Bacteria	*SMWR01 (UBA9160)*	50.42%	6.45%	2,672,116	2,872	Medium
	**Archaea**	***Methanolobus (Methanosarcinales)***	**92.81%**	**1.96%**	**2,489,475**	**2,669**	**Medium**
	Archaea	*UBA7939 (Methanosarcinales)*	87.58%	0.65%	2,224,045	2,673	Medium
**Station H3**	Bacteria	*Thiobacillus (Burkholderiales)*	100%	0.48%	3,269,799	3,337	Medium
	Bacteria	*Sulfuricella (Nitrosomonadales)*	99.29%	0.98%	2,882,075	2,947	Medium
	Bacteria	*Gemmatimonadetes*	93.20%	4.95%	3,005,382	2,914	Medium
	Bacteria	*UBA9214 (Thiohalobacterales)*	77.45%	1.90%	2,684,927	2,952	Medium
	Bacteria	*Methylophagaceae (Nitrosococcales)*	72.41%	0.00%	2,112,498	2,215	Medium
	Bacteria	*UBA8639 (Nitrospirales)*	58.53%	4.02%	1,749,825	1,957	Medium
	Bacteria	*Ignavibacterium (Ignavibacteriales)*	57.37%	7.94%	1,714,699	1,870	Medium
	Bacteria	*CG2-30-66-27 (MBNT15)*	55.57%	0.84%	1,184,603	1,340	Medium
	Bacteria	*SPDF01 (Gemmatimonadales)*	53.44%	7.14%	1,471,751	1,636	Medium
	Bacteria	*BM004 (Desulfobulbales)*	53.06%	1.81%	1,085,505	1,216	Medium
	Archaea	*UBA10834 (Thermoplasmata)*	83.37%	1.20%	1,375,321	1,451	Medium
	**Archaea**	***Methanolobus (Methanosarcinales)***	**66.74%**	**1.31%**	**1,070,939**	**1,261**	**Medium**
**Station H5**	Bacteria	*Methylophagaceae (Nitrosococcales)*	99.18%	0.88%	2,928,202	2,798	Medium
	Bacteria	*Sulfuricella (Nitrosomonadales)*	95.50%	2.84%	3,050,167	3,151	Medium
	Bacteria	*M0040 (Desulfuromonadales)*	89.22%	0.65%	3,047,111	3,157	Medium
	Bacteria	*Sulfurimonas (Campylobacterales)*	79.44%	3.88%	1,756,086	1,894	Medium
	Bacteria	*UBA6164 (Gracilibacteria)*	76.99%	2.36%	1,161,215	2,152	Medium
	Bacteria	*UBA9214 (Thiohalobacterales)*	76.14%	7.30%	2,418,493	2,607	Medium
	Bacteria	*BM004 (Desulfobulbales)*	70.72%	1.52%	1,624,494	1,775	Medium
	Bacteria	*Lutibacter (Flavobacteriales)*	65.18%	3.28%	1,960,362	2,061	Medium
	Bacteria	*UBA2258*	62.76%	1.65%	1,130,384	1,198	Medium
	**Archaea**	***Methanolobus (Methanosarcinales)***	**88.89%**	**1.31%**	**2,579,227**	**2,760**	**Medium**
	**Archaea**	***Methanolobus (Methanosarcinales)***	**62.58%**	**0%**	**1,291,140**	**1,362**	**Medium**
	Archaea	*SMTZ1-45 (Thorarchaeales)*	54.35%	0.47%	677,230	810	Medium

Supplementary Table 4. Analysis of the *mts* genes within the whole genome sequences of *Methanolobus* strains available on JGI and NCBI databases. *mtsA* and *mtsB* have < %38 identity, whilst *mtsF* had < %56 identity at the amino acid level.

	Strain name	Genome/Tax ID	*mtsA*	*mtsB*	*mtsD*	*mtsF*	*mtsH*
JGI	***M. bombayensis*** **DSM 7082**	2913348608	-	-	80%	-	69%
	M. profundi **Mob M**	2642422540	-	-	-	-	-
	M. psychrophilus **R15**	2519103099	-	-	-	-	-
	M. tindarius **DSM 2278**	2515075008	-	-	-	-	-
	M. vulcani **B1d**	8001971949	-	-	80%	-	69%
	M. vulcani **PL 12/M**	2642422561	-	-	-	-	-
	M. zinderi **DSM 21339**	2883920249	-	-	81%	-	69%
NCBI	***M. tindarius***	1090322	-	-	-	-	-
	M. psychrophilus	420950	-	-	-	-	-
	M. vulcani	38026	-	-	80%	-	68%
	M. chelungpuianus	502115	-	-	-	-	-
	M. halotolerans	2052935	-	-	78%	-	66%
	M. bombayensis	38023	-	-	79%	-	69%
	M. zinderi	536044	-	-	80%	-	69%
	M. psychrotolerans	1874706	-	-	-	-	-
	M. profundi	487685	-	-	-	-	-
